# Comparison of Different Lymph Node Staging Schemes for Predicting Survival Outcomes in Node-Positive Endometrioid Endometrial Cancer Patients

**DOI:** 10.3389/fmed.2021.688535

**Published:** 2021-07-09

**Authors:** Xi-Lin Yang, Nan Huang, Ming-Ming Wang, Hua Lai, Da-Jun Wu

**Affiliations:** ^1^Department of Radiation Oncology, Chengdu Women's and Children's Central Hospital, School of Medicine, University of Electronic Science and Technology of China, Chengdu, China; ^2^Department of Oncology, The First Affiliated Hospital of Chongqing Medical University, Chongqing, China; ^3^Department of Radiology, Chengdu Women's and Children's Central Hospital, School of Medicine, University of Electronic Science and Technology of China, Chengdu, China

**Keywords:** endometrioid endometrial cancer, lymph node ratio, log odds of positive lymph nodes, prognosis, SEER

## Abstract

**Objective:** To compare the prognostic predictive performance of six lymph node (LN) staging schemes: American Joint Committee on Cancer (AJCC) N stage, number of retrieved lymph nodes (NRLN), number of positive lymph nodes (NPLN), number of negative lymph nodes (NNLN), lymph node ratio (LNR), and log odds of positive lymph nodes (LODDS) among node-positive endometrioid endometrial cancer (EEC) patients.

**Methods:** A total of 3,533 patients diagnosed with node-positive EEC between 2010 and 2016 from the Surveillance, Epidemiology, and End Results (SEER) database were retrospectively analyzed. We applied X-tile software to identify the optimal cutoff value for different staging schemes. Univariate and multivariate Cox regression models were used to assess the relationships between different LN schemes and survival outcomes [disease-specific survival (DSS) and overall survival (OS)]. Moreover, Akaike information criterion (AIC) and Harrell concordance index (C-index) were used to evaluate the predictive performance of each scheme in both continuous and categorical patterns.

**Results:** N stage (N1/N2) was not an independent prognostic factor for node-positive EEC patients based on multivariate analysis (DSS: *p* = 0.235; OS: *p* = 0.145). Multivariate model incorporating LNR demonstrated the most superior goodness of fit regardless of continuous or categorical pattern. Regarding discrimination power of the models, LNR outperformed other models in categorical pattern (OS: C-index = 0.735; DSS: C-index = 0.737); however, LODDS obtained the highest C-index in continuous pattern (OS: 0.736; DSS: 0.739).

**Conclusions:** N stage (N1/N2) was unable to differentiate the prognosis for node-positive EEC patients in our study. However, LNR and LODDS schemes seemed to have a better predictive performance for these patients than other number-based LN schemes whether in DSS or OS, which revealed that LNR and LODDS should be more helpful in prognosis assessment for node-positive EEC patients than AJCC N stage.

## Introduction

Endometrial cancer (EC) was one of the most common gynecological malignancies, with 382,069 new cases expected worldwide in 2018 (1). Histologically, 80% of the EC patients belonged to type I EC, also known as endometrioid adenocarcinoma. The remaining 20% of the EC patients were type II EC that was more aggressive in nature (2). The majority of the endometrioid endometrial cancer (EEC) patients were diagnosed at an early stage with a relatively promising prognosis (3). However, the 5-year disease-specific survival (DSS) for node-positive EEC patients ranged from 44 to 77%, which suggested that considerable heterogeneity existed among these patients (4, 5). As a result, a more predictive lymph node (LN) staging system was needed for node-positive EEC patients.

It was noted that LN status has been one of the most significant prognostic factors for EEC patients (6). Recently, node-positive EC was divided into N1 (pelvic LN involvement) and N2 (para-aortic LN involvement with or without pelvic LN involvement) (7). This new N staging principle made sense from the perspective of regional LN drainage, as the pelvic LN basin harbored almost all of the uterine LN drainage while the para-aortic LN involvement might imply further progression (8, 9). However, this staging system did not take LN number or ratio into consideration, and previous studies have revealed that a higher number of positive lymph nodes (NPLN) or lymph node ratio (LNR) was related to more dreadful survival for EEC patients (10–12). So, it is necessary to further explore the prognostic significance of these LN schemes.

LNR, defined as NPLN divided by the number of retrieved lymph nodes (NRLN), has been found useful in evaluating the prognosis in node-positive EEC patients (4, 13, 14). Log odds of positive lymph nodes (LODDS) was a nascent prognostic parameter that has currently been used to assess the prognosis in various malignancies (15–18). To the best of our knowledge, little clinical evidence has recommended the most suitable LN scheme to predict the prognosis for node-positive EEC patients. Meanwhile, the prognostic role of LODDS in EEC patients has not been established in previous studies. Therefore, we conducted this study to compare the predictive performance of six different LN schemes in node-positive EEC patients.

## Methods

### Data Source and Population Selection

The data were collected from the Surveillance, Epidemiology, and End Results (SEER) database consisting of ~34.6% of the cancer population in the United States (19). We extracted the relevant data through SEER^*^Stat software (version 8.4.6; http://seer.cancer.gov/seerstat/) with permission to access the database (Authorization Code: 17548-Nov2020). Since the data available in this program were anonymous, the requirement for informed consent was waived. We created a case listing including all EEC patients (histological codes: 8080/3, 8081/3, 8082/3, 8083/3) diagnosed between 2010 and 2016 based on the third edition of the International Classification of Disease Oncology (ICD-O-3). LN metastasis was defined as macrometastasis or micrometastasis in the sampling LN according to the pathological findings in the SEER database. Patients who met any of the following criteria would be excluded: (1) Patients with insufficient information regarding LN status, clinical–pathological characteristics, surgical, and follow-up treatment; (2) Patients without LN metastasis (N0 stage); (3) Patients without LN retrieving during the surgery; (4) T4 (bladder mucosa or rectal mucosa was invaded) or M1 (distant metastasis); (5) Patients without hysterectomy; (6) Age <18 years; (7) Patients died from surgical complications (survival time <1 month); (8) Patients with malignant tumor history. Age at diagnosis was divided into two groups (<65, ≥65) according to previous studies (20, 21).

### Number of Retrieved Lymph Nodes, Number of Positive Lymph Nodes, Number of Negative Lymph Nodes, Lymph Node Ratio, and Log Odds of Positive Lymph Nodes Classification

NRLN represented the total number of retrieved LNs during the surgery. NPLN was defined as the number of positive LNs. Number of negative lymph nodes (NNLN), known as the number of negative LNs among the retrieved LNs, could be estimated as NNLN = NRLN – NPLN. LNR was calculated as NPLN/NRLN. LODDS was defined as log_e_ [(NPLN + 0.5)/(NNLN + 0.5)], where 0.5 was added to both the denominator and numerator to avoid singularity (22). We trichotomized these five continuous variables (NRLN, NPLN, NNLN, LNR, LODDS) *via* X-tile software (version 3.6.1; Yale University, New Haven, CT, USA) once maximal chi-square value was reached, which was considered to represent the greatest difference in prognosis prediction among the subgroups (23, 24). In our study, NRLN was divided into three subgroups: NRLN1 (1–5), NRLN2 (6–14), NRLN3 (≥15). NPLN was divided into NPLN1 (1), NPLN2 (2–6), and NPLN3 (≥7). NNLN was categorized as NNLN1 (<5), NNLN2 (5–13), and NNLN3 (≥14). LNR was classified into LNR1 (0.01–0.15), LNR2 (0.16–0.66), and LNR3 (≥0.67). LODDS was divided into three subgroups: −4.07 < LODDS1 ≤-1.44, −1.44 < LODDS2 ≤0.49, and 0.49 < LODDS3 ≤4.32.

### Statistical Analysis

We conducted a two-step Cox proportional hazard regression analysis to illustrate the association between the different LN schemes and OS. Firstly, univariate Cox regression analysis was performed to select the possible prognostic factors (*p* < 0.100), which would be included in the subsequent multivariate Cox regression analysis. Secondly, we conducted six multivariate Cox regression models with each model incorporating one LN scheme: Model 1 (N), Model 2 (NRLN), Model 3 (NPLN), Model 4 (NNLN), Model 5 (LNR), and Model 6 (LODDS). In order to maintain the authenticity of original data, the continuous pattern of the data was processed in the same way. Predictive performance of different LN models was assessed from multiple perspectives. AIC was adopted to evaluate the models' goodness of fit (25). Moreover, C-index was calculated to appraise the discrimination power of the LN models (26).

The correlations between the continuous patterns of NRLN, NPLN, NNLN, LNR, and LODDS were visualized and compared with scatter plot and Pearson correlation coefficient (r), respectively. Sensitivity analysis using DSS as endpoint was then performed. All analyses were performed using R software (version 3.6.1; http://www.r-project.org) and IBM SPSS 22.0 (Armonk, NY). A two-tailed *p* < 0.05 is recognized as statistically significant.

## Results

### Patient Characteristics and Survival Analysis

A total of 3,533 EEC patients remained in our final study cohort. The selection process was shown in detail (Supplementary Table 1). We summarized the baseline clinical–pathological and demographic characteristics in Table 1. The median NRLN was 16 [interquartile range (IQR): 9–25], and mean NRLN was 18.47 ± 12.57. Similarly, the median NPLN and mean NPLN were 2 (IQR: 1–4) and 3.49 ± 4.68, respectively. The mean NNLN was 14.98 ± 11.67, and the median NNLN was 13 (IQR: 6–21). The mean LNR and median LNR were 0.15 (IQR: 0.08–0.33) and 0.26 ± 0.26. The mean LODDS was −1.29 ± 1.26, and the median LODDS was −1.53 (IQR: −2.20 to −0.59). More than half of the NRLN in eligible patients exceeded 15, which further ensured the accuracy of LN sampling.

**Table 1:** Clinical and pathological characteristics and survival analysis of patients.

**Variable**	**Number (%)**	**5-year OS**		**5-year DSS**	
		**% (95% CI)**	**Log-rank *p*-value**	**% (95% CI)**	**Log-rank *p*-value**
**Age**			*p* < 0.001		*p* < 0.001
<65	1,926 (54.5%)	68.6 (65.9–71.5)		71.1 (68.4–74.0)	
≥65	1,607 (45.5%)	49.2 (46.1–52.6)		52.5 (49.3–55.9)	
**Race**			*p* < 0.001		*p* < 0.001
White	2,715 (76.8%)	61.5 (59.1–64.0)		64.7 (62.4–67.1)	
Black	426 (12.1%)	43.9 (37.8–50.9)		46.5 (40.2–53.8)	
Other	392 (11.1%)	64.6 (58.3–71.5)		65.9 (59.6–72.8)	
**Marital status**			*p* < 0.001		*p* < 0.001
Married	1,741 (49.3%)	63.9 (60.9–67.0)		66.2 (63.3–69.4)	
Unmarried	1,792 (50.7%)	55.9 (52.9–59.0)		59.4 (56.4–62.5)	
**Grade**			*p* < 0.001		*p* < 0.001
I	533 (15.1%)	78.1 (73.3–83.3)		81.5 (76.9–86.3)	
II	1,044 (29.5%)	75.3 (71.8–78.9)		78.1 (74.6–81.6)	
III	1,386 (39.1%)	46.5 (43.1–50.1)		49.3 (45.9–53.0)	
IV	570 (16.1%)	45.8 (40.5–51.7)		47.8 (42.4–53.9)	
**T stage**			*p* < 0.001		*p* < 0.001
T1	1,766 (50.0%)	70.7 (67.9–73.6)		73.7 (71.0–76.6)	
T2	642 (18.2%)	59.2 (54.4–64.5)		61.8 (57.0–67.0)	
T3	1,125 (31.8%)	43.2 (39.4–47.2)		45.9 (42.1–50.1)	
**Tumor size**			*p* < 0.001		*p* < 0.001
<4 cm	851 (24.1%)	65.6 (61.4–70.2)		67.2 (62.9–71.7)	
≥4 cm	2,113 (59.8%)	56.3 (53.4–59.2)		59.5 (56.7–62.5)	
Unknown	569 (16.1%)	63.2 (58.5–68.1)		67.0 (62.4–71.9)	
**Surgery type**			*p* < 0.001		*p* < 0.001
Total	3,006 (85.1%)	61.0 (58.7–63.4)		63.8 (61.5–66.2)	
Radical	527 (14.9%)	52.8 (47.5–58.7)		56.6 (51.3–62.5)	
**Radiation**			*p* < 0.001		*p* < 0.001
Yes	2,017 (57.1%)	66.2 (63.4–69.1)		68.6 (65.9–71.5)	
No	1,516 (42.9%)	51.5 (48.4–54.9)		55.0 (51.8–58.4)	
**Chemo**			*p* < 0.001		*p* < 0.001
Yes	2,769 (78.4%)	62.0 (59.6–64.6)		64.5 (62.0–67.0)	
No/Unknown	764 (21.6%)	51.7 (47.5–56.3)		56.2 (51.9–60.8)	
**N stage**			*p* < 0.001		*p* < 0.001
N1	2,241 (63.4%)	62.8 (60.1–65.5)		66.0 (63.3–68.7)	
N2	1,292 (36.6%)	54.8 (51.3–58.5)		57.4 (53.9–61.1)	
**NRLN**			*p* < 0.001		*p* < 0.001
NRLN1	438 (12.4%)	42.7 (36.5–50.5)		45.7 (39.3–53.2)	
NRLN2	1,101 (31.2%)	60.0 (56.3–64.0)		63.2 (59.5–67.2)	
NRLN3	1,994 (56.4%)	63.0 (60.3–65.9)		65.8 (63.1–68.6)	
**NPLN**			*p* < 0.001		*p* < 0.001
NPLN1	1,371 (38.8%)	66.9 (63.7–70.2)		69.5 (66.3–72.8)	
NPLN2	1,737 (49.2%)	59.5 (56.5–62.7)		62.7 (59.6–65.8)	
NPLN3	425 (12.0%)	38.0 (32.2–44.9)		40.9 (34.8–48.1)	
**NNLN**			*p* < 0.001		*p* < 0.001
NNLN1	660 (18.7%)	41.3 (36.4–46.9)		44.4 (39.3–50.2)	
NNLN2	1,190 (33.7%)	58.4 (54.7–62.3)		61.3 (57.6–65.2)	
NNLN3	1,683 (47.6%)	67.4 (64.5–70.4)		70.1 (67.2–73.1)	
**LNR**			*p* < 0.001		*p* < 0.001
LNR1	1,785 (50.5%)	69.8 (67.1–72.7)		72.4 (69.7–75.2)	
LNR2	1,391 (39.4%)	53.2 (49.6–57.0)		56.4 (52.8–60.3)	
LNR3	357 (10.1%)	31.4 (25.6–38.6)		33.9 (27.8–41.3)	
**LODDS**			*p* < 0.001		*p* < 0.001
LODDS1	1,869 (52.9%)	69.6 (66.9–72.4)		72.1 (69.5–74.9)	
LODDS2	1,294 (36.6%)	52.3 (48.6–56.3)		55.7 (51.9–59.8)	
LODDS3	370 (10.5%)	32.2 (26.5–39.2)		34.6 (28.7–41.9)	

*OS, overall survival; DSS, disease-specific survival; NRLN, number of retrieved lymph nodes; NPLN, number of positive lymph nodes; NNLN, number of negative lymph nodes; LNR, lymph node ratio; LODDS, log odds of positive lymph nodes; CI, confidence interval*.

**Figure 1:**
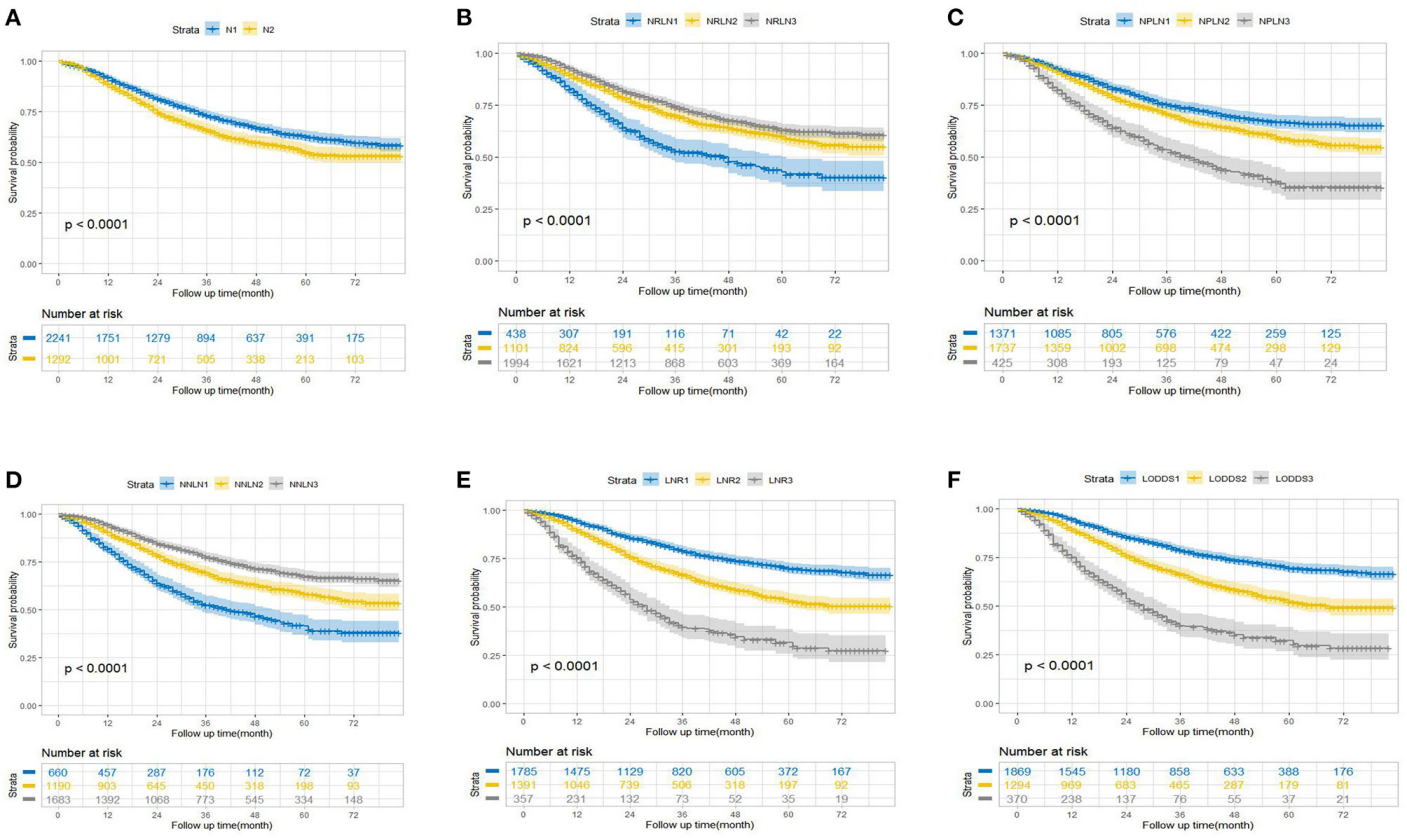
Kaplan–Meier estimates of overall survival according to **(A)** N, **(B)** NRLN, **(C)** NPLN, **(D)** NNLN, **(E)** LNR, and **(F)** LODDS staging systems. NRLN, number of retrieved lymph nodes; NPLN, number of positive lymph nodes; NNLN, number of negative lymph nodes; LNR, lymph node ratio; LODDS, log odds of positive lymph nodes.

The survival probability for node-positive EEC patients increased as the increment of the value of NRLN and NNLN. For example, the 5-year OS increased from 42.7% (36.5–50.5%) for NRLN1 to 63.0% (60.3–65.9%) for NRLN3. Furthermore, this also can be validated by the restricted cubic spline analysis that mortality risk decreased as NRLN increased (Supplementary Figure 1A). However, the survival probability for node-positive EEC patients decreased as the increment of NPLN, LNR, and LODDS. For instance, the 5-year DSS was reduced from 72.1% (69.5–74.9%) for LODDS1 to 34.6% (28.7–41.9%) for LODDS3 (*P* < 0.001). Still, as shown in Supplementary Figure 1E, mortality risk increased as LODDS increased. The Kaplan–Meier curves for OS and DSS stratified by different LN schemes were presented in Figure 1 and Supplementary Figure 2, respectively. Six multivariate Cox models were set up, with each model incorporating one LN scheme and other prognostic factors identified in the univariate Cox regression analysis (Supplementary Table 2), which were exhibited as Model 1 (N), Model 2 (NRLN), Model 3 (NPLN), Model 4 (NNLN), Model 5 (LNR), and Model 6 (LODDS) in Table 2. We found that N stage (N1/N2) was not significantly associated with survival outcomes regardless of DSS (*p* = 0.253) or OS (*p* = 0.145).

**Table 2:** Multivariable Cox regression analysis (Models 1–6) of prognostic predictors for OS.

	**Model 1 (N)**		**Model 2 (NRLN)**		**Model 3 (NPLN)**		**Model 4 (NNLN)**		**Model 5 (LNR)**		**Model 6 (LODDS)**	
**Variable**	**HR (95% CI)**	***p*-value**	**HR (95% CI)**	***p*-value**	**HR (95% CI)**	***p*-value**	**HR (95% CI)**	***p*-value**	**HR (95% ci)**	***p-*value**	**HR (95% CI)**	***p*-value**
**Age**												
<65	1		1		1		1		1		1	
≥65	1.651 (1.450, 1.879)	*p* < 0.001	1.670 (1.467, 1.901)	*p* < 0.001	1.648 (1.447, 1.876)	*p* < 0.001	1.671 (1.467, 1.903)	*p* < 0.001	1.684 (1.479, 1.918)	*p* < 0.001	1.680 (1.475, 1.913)	*p* < 0.001
**Race**												
White	1		1		1		1		1		1	
Black	1.219 (1.023, 1.453)	*p* = 0.027	1.210 (1.015, 1.442)	*p* = 0.034	1.191 (0.999, 1.419)	*p* = 0.052	1.154 (0.968, 1.377)	*p* = 0.111	1.122 (0.940, 1.339)	*p* = 0.201	1.125 (0.943, 1.342)	*p* = 0.192
Other	0.864 (0.692, 1.080)	*p* = 0.199	0.858 (0.687, 1.072)	*p* = 0.177	0.881 (0.705, 1.101)	*p* = 0.264	0.842 (0.674, 1.052)	*p* = 0.130	0.840 (0.672, 1.050)	*p* = 0.105	0.841 (0.678, 1.051)	*p* = 0.127
**Marital status**												
Married	1		1		1		1		1		1	
Unmarried	1.106 (0.971, 1.258)	*p* = 0.129	1.090 (0.958, 1.241)	*p* = 0.191	1.109 (0.974, 1.262)	*p* = 0.118	1.088 (0.955, 1.238)	*p* = 0.204	1.107 (0.973, 1.260)	*p* = 0.123	1.100 (0.966, 1.252)	*p* = 0.151
**Grade**												
I	1		1		1		1		1		1	
II	1.218 (0.925, 1.603)	*p* = 0.160	1.237 (0.939, 1.629)	*p* = 0.130	1.197 (0.909, 1.576)	*p* = 0.200	1.239 (0.941, 1.631)	*p* = 0.127	1.228 (0.933, 1.617)	*p* = 0.142	1.210 (0.919, 1.592)	*p* = 0.175
III	2.702 (2.101, 3.475)	*p* < 0.001	2.705 (2.103, 3.479)	*p* < 0.001	2.615 (2.032, 3.365)	*p* < 0.001	2.666 (2.072, 3.428)	*p* < 0.001	2.581 (2.006, 3.321)	*p* < 0.001	2.556 (1.986, 3.289)	*p* < 0.001
IV	2.880 (2.190, 3.787)	*p* < 0.001	2.958 (2.249, 3.891)	*p* < 0.001	2.781 (2.114, 3.659)	*p* < 0.001	2.934 (2.231, 3.858)	*p* < 0.001	2.866 (2.180, 3.769)	*p* < 0.001	2.834 (2.155, 3.726)	*p* < 0.001
**T stage**												
T1	1		1		1		1		1		1	
T2	1.262 (1.052, 1.513)	*p* = 0.012	1.257 (1.048, 1.507)	*p* = 0.014	1.249 (1.041, 1.499)	*p* = 0.017	1.225 (1.022, 1.470)	*p* = 0.028	1.217 (1.015, 1.460)	*p* = 0.034	1.222 (1.019, 1.465)	*p* = 0.031
T3	2.127 (1.837, 2.464)	*p* < 0.001	2.058 (1.777, 2.384)	*p* < 0.001	2.015 (1.736, 2.338)	*p* < 0.001	1.950 (1.682, 2.262)	*p* < 0.001	1.798 (1.546, 2.091)	*p* < 0.001	1.796 (1.544, 2.089)	*p* < 0.001
**Tumor size**												
<4cm	1		1		1		1		1		1	
≥4cm	1.237 (1.051, 1.456)	*p* = 0.011	1.224 (1.039, 1.441)	*p* = 0.015	1.218 (1.034, 1.434)	*p* = 0.018	1.207 (1.025, 1.422)	*p* = 0.024	1.216 (1.033, 1.432)	*p* = 0.019	1.213 (1.030, 1.428)	*p* = 0.021
Unknown	1.063 (0.862, 1.311)	*p* = 0.567	1.075 (0.872, 1.325)	*p* = 0.498	1.042 (0.845, 1.285)	*p* = 0.701	1.063 (0.862, 1.311)	*p* = 0.564	1.020 (0.827, 1.258)	*p* = 0.855	1.025 (0.831, 1.264)	*p* = 0.820
**Surgery ty*****p*****e**												
Total	1		1		1		1		1		1	
Radical	1.189 (1.011, 1.399)	*p* = 0.036	1.215 (1.033, 1.430)	*p* = 0.019	1.182 (1.005, 1.391)	*p* = 0.043	1.228 (1.043, 1.445)	*p* = 0.014	1.204 (1.024, 1.417)	*p* = 0.025	1.208 (1.027, 1.422)	*p* = 0.023
**Radiation**												
Yes	1		1		1		1		1		1	
No	1.567 (1.373, 1.787)	*p* < 0.001	1.550 (1.359, 1.767)	*p* < 0.001	1.545 (1.354, 1.763)	*p* < 0.001	1.553 (1.361, 1.770)	*p* < 0.001	1.505 (1.319, 1.717)	*p* < 0.001	1.511 (1.324, 1.724)	*p* < 0.001
**Chemo**												
Yes	1		1		1		1		1		1	
No/Unknown	1.489 (1.287, 1.722)	*p* < 0.001	1.475 (1.276, 1.706)	*p* < 0.001	1.504 (1.300, 1.741)	*p* < 0.001	1.462 (1.264, 1.691)	*p* < 0.001	1.506 (1.302, 1.742)	*p* < 0.001	1.502 (1.298, 1.737)	*p* < 0.001
**N stage**												
N1	1											
N2	1.078 (0.948, 1.226)	*p* = 0.253										
**NRLN**												
NRLN1			1									
NRLN2			0.667 (0.552, 0.806)	*p* < 0.001								
NRLN3			0.590 (0.494, 0.703)	*p* < 0.001								
**NPLN**												
NPLN1					1							
NPLN2					1.135 (0.983, 1.311)	*p* = 0.083						
NPLN3					1.595 (1.321, 1.926)	*p* < 0.001						
**NNLN**												
NNLN1							1					
NNLN2							0.643 (0.547, 0.756)	*p* < 0.001				
NNLN3							0.507 (0.432, 0.596)	*p* < 0.001				
**LNR**												
LNR1									1			
LNR2									1.490 (1.294, 1.716)	*p* < 0.001		
LNR3									2.624 (2.172, 3.169)	*p* < 0.001		
**LODDS**												
LODDS1											1	
LODDS2											1.492 (1.296, 1.719)	*p* < 0.001
LODDS3											2.566 (2.131, 3.090)	*p* < 0.001

*OS, overall survival; NRLN, number of retrieved lymph nodes; NPLN, number of positive lymph nodes; NNLN, number of negative lymph nodes; LNR, lymph node ratio; LODDS, log odds of positive lymph nodes; CI, confidence interval*.

### Prognostic Performance Among Number of Retrieved Lymph Nodes, Number of Positive Lymph Nodes, Number of Negative Lymph Nodes, Lymph Node Ratio, and Log Odds of Positive Lymph Nodes

We excluded N stage (N1/N2) from the comparison of prognostic performance seeing that N stage did not show any prognostic ability in Model 1. The predictive performance of the remaining five Cox models was assessed (Table 3). Model 5 (LNR) showed the lowest AIC (AIC: 14,453.81) and the highest C-index value (C-index: 0.735) among the five models in categorical pattern. Subsequently, we compared these five models in continuous pattern, which revealed that Model 5 (LNR) still had the lowest AIC (AIC: 14,456.17), but the highest C-index value was obtained by Model 6 (LODDS) (C-index: 0.736). Interestingly, we observed LODDS and LNR had almost identical C-index values in OS regardless of continuous (LODDS vs. LNR: 0.736 vs. 0.735) or categorical pattern (LODDS vs. LNR: 0.734 vs. 0.735). Therefore, it would be reckless to simply conclude LNR or LODDS as the best-performance model in predicting OS for EEC patients. Meanwhile, the C-index value for all the five models exceeded 0.70, which demonstrated a considerably good predictive accuracy (27).

**Table 3:** Predictive performance of different LN schemes.

	**OS**		**DSS**	
**Model**	**AIC**	**C-index (95% CI)**	**AIC**	**C-index (95% CI)**
**Categorical**				
Model 2 (NRLN)	14,528.27	0.723 (0.707–0.739)	12,935.51	0.725 (0.709–0.741)
Model 3 (NPLN)	14,536.77	0.724 (0.709–0.740)	12,940.20	0.728 (0.712–0.743)
Model 4 (NNLN)	14,488.16	0.729 (0.714–0.745)	12,898.99	0.731 (0.715–0.746)
Model 5 (LNR)	14,453.81	0.735 (0.719–0.751)	12,863.51	0.737 (0.722–0.753)
Model 6 (LODDS)	14,453.98	0.734 (0.718–0.750)	12,864.37	0.737 (0.721–0.753)
**Continuous**				
Model 2 (NRLN)	14,537.68	0.722 (0.706–0.737)	12,941.22	0.724 (0.708–0.739)
Model 3 (NPLN)	14,548.50	0.733 (0.718–0.749)	12,951.54	0.726 (0.710–0.742)
Model 4 (NNLN)	14,508.62	0.726 (0.710–0.741)	12,914.70	0.728 (0.712–0.743)
Model 5 (LNR)	14,456.17	0.735 (0.719–0.750)	12,866.34	0.738 (0.722–0.754)
Model 6 (LODDS)	14,457.38	0.736 (0.720–0.751)	12,869.48	0.739 (0.723–0.754)

*OS, overall survival; DSS, disease-specific survival; NRLN, number of retrieved lymph nodes; NPLN, number of positive lymph nodes; NNLN, number of negative lymph nodes; LNR, lymph node ratio; LODDS, log odds of positive lymph nodes; CI, confidence interval; AIC, Akaike information criterion*.

### Correlation Among Different Lymph Node Schemes

**Figure 2:**
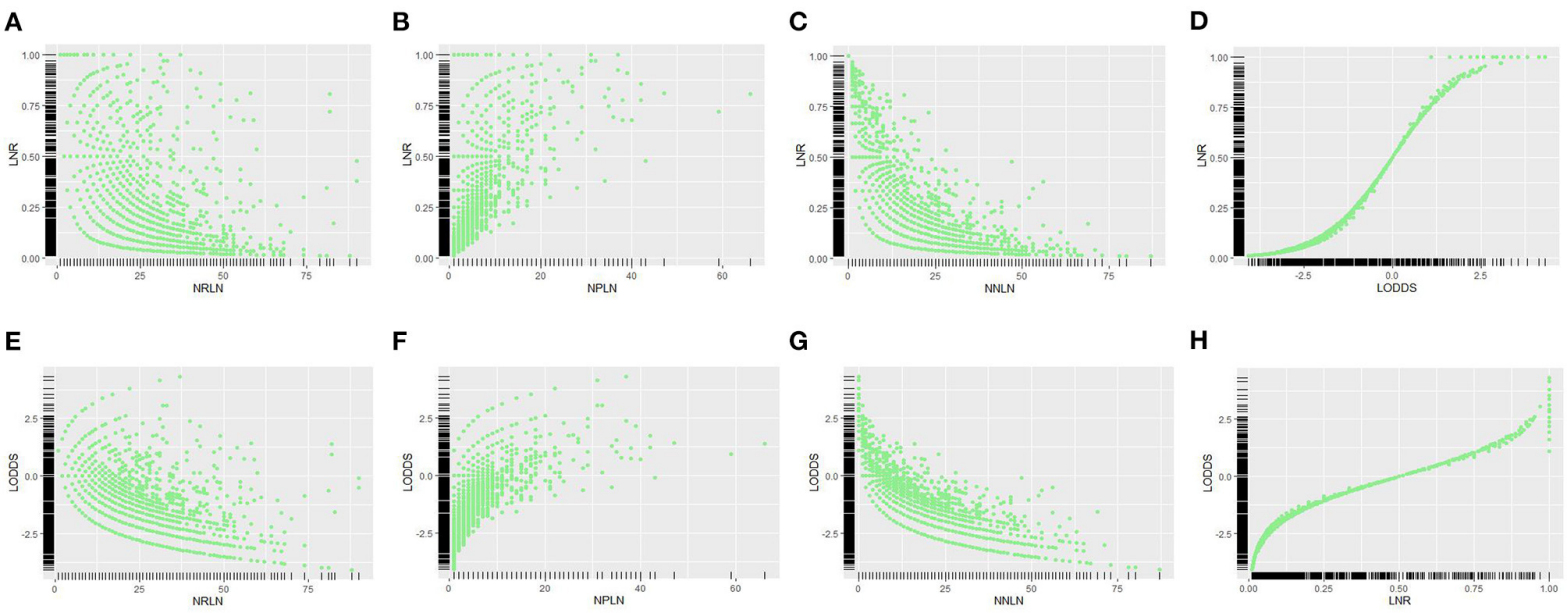
Scatter plots of the relationship between LNR vs. **(A)** NRLN, **(B)** NPLN, **(C)** NNLN, and **(D)** LODDS. The relationship between LODDS vs. **(E)** NRLN, **(F)** NPLN, **(G)** NNLN, and **(H)** LNR was described. NRLN, number of retrieved lymph nodes; NPLN, number of positive lymph nodes; NNLN, number of negative lymph nodes; LNR, lymph node ratio; LODDS, log odds of positive lymph nodes.

Scatter plot was used to visualize the relationship among different LN schemes, which demonstrated that LNR was more correlated with LODDS than other LN schemes (*r* = 0.913 vs. *r* = 0.148, *r* = 0.181 and *r* = 0.341; all *p* < 0.001). Besides, LNR and LODDS were positively correlated with NPLN (Figures 2B,F); however, they were negatively correlated with NRLN (Figures 2A,E) and NNLN (Figures 2C,G). Notably, LNR and LODDS differentiate the prognosis for patients with the same NPLN even when NPLN ≤5 (Figures 2B,F). Similarly, LNR and LODDS could remain heterogeneous even when NRLN is small (Figures 2A,E). In addition, LNR was positively associated with LODDS, though the correlation between them was not precisely linear (Figures 2D,H). As shown in Figure 2D, the curve became much steeper when LNR ≤0.2 or LNR ≥0.8, which suggested that LNR increased much slower than LODDS in this range. Therefore, LODDS might perform better in predicting prognosis when LNR was either low or high.

### Sensitivity Analysis

We used the same approach to process DSS. Multivariate Cox analysis revealed that Model 1 (N) was not an independent risk factor for DSS either (Supplementary Table 3). In the assessment of predictive models, Model 5 (LNR) remained the best performer in the evaluation of goodness of fit, which had the lowest AIC in both continuous (AIC: 12,866.34) and categorical patterns (AIC: 12,863.51; Table 3). Similarly, LNR and LODDS still had the resembling discrimination power regardless of continuous pattern (C-index: LNR vs. LODDS = 0.738 vs. 0.739) or categorical pattern (C-index: LNR vs. LODDS = 0.737 vs. 0.737; Table 3).

## Discussion

The updated N staging system based on anatomic location was considered the optimal way to predict the prognosis for node-positive EC patients currently, but this LN scheme mingled EEC patients with non-EEC patients, which overlooked the fact that EEC patients had more promising outcomes than non-EEC patients (28). In our study, the American Joint Committee on Cancer (AJCC) N stage was meaningless in prognosis prediction for node-positive EEC patients after surgery, which was consistent with the previous study showing that EEC patients with isolated pelvic LN metastasis shared a similar prognosis with EEC patients with isolated para-aortic LN metastasis (29).

In terms of NRLN, previous study showed that more than 20 retrieved LNs during the surgery could benefit the survival of EEC patients; however, this study included both the node-positive and node-negative EEC patients (30). In our study, the optimal NRLN was 15, and increasing NRLN was linked to better DSS and OS, which deserved to be further evaluated. We have noticed that the NPLN scheme has been used to assess prognosis for EEC patients, which revealed that patients with only one LN involvement outperformed patients with multiple LN involvement regarding OS and DSS (12). However, not only the NPLN scheme could be affected by NRLN during the surgery but also the pathological examination procedure might have an impact on the result, which could lead to miscounting of NPLN. Similarly, the NNLN scheme shared the same concerns with NPLN (18).

The LNR scheme covered the information of both NPLN and NRLN, which was able to surmount the limitations of the above number-based LN schemes theoretically. Numerous studies had established the prognostic role of LNR scheme in other gynecological oncologies (18, 31, 32). In EC patients, the LNR scheme was deemed to be a novel independent prognostic index after surgery (4, 10, 13). However, this scheme still had its limitations. Firstly, LNR scheme was genetically not suitable for distinguishing the survival among node-negative EEC patients. Secondly, patients with different NRLN and NPLN might share identical LNRs, which brought up the obvious question on whether EEC patients with one positive LN out of one retrieved LN performed equally in OS and DDS compared to patients with five positive LNs out of five retrieved LNs. As a nascent parameter, LODDS scheme could make up for LNR, which has been thoroughly investigated in various malignancies and showed superior prognostic predictive power than the LNR scheme (15–17, 24, 33, 34).

N staging (Model 1) was washed out for not being an independent prognostic factor for survival, which suggested that more aggressive adjuvant therapy for N2 patients than N1 patients was debatable. In the current study, node-positive EEC patients with more NRLN, more NNLN, less NPLN, lower LNR, and lower LODDS performed better in DSS and OS (Table 2, Supplementary Table 3), which implied that more aggressive adjuvant therapy and closer follow-up might be arranged for these patients. Scatter plots were conducted to visualize the reciprocal correlations among these LN schemes, which revealed the tightest relationship between LNR and LODDS (Figures 2D,H). This was not hard to explain given that these two schemes were both ratio-based LN schemes.

Some strengths and limitations of this study should be noted. High-volume data from the SEER registry ensured the sophisticated generalizability and universality of this study. On the other hand, each multivariate Cox regression model was assessed not just in discrimination power but also in goodness of fit, which resulted in the addition of comprehensiveness and rigorousness to this study. Moreover, we appraised the predictive power of these models in continuous and categorical patterns to avoid data distortion. Last but not least, ratio-based LN schemes were demonstrated to be more useful in prognosis prediction for node-positive EEC patients, which provided an alternative way to assess the survival outcomes for these patients. However, several limitations in our study also needed to be noted. Firstly, as a retrospective study, selection bias was difficult to avoid, and prospective studies are welcome in the future. Secondly, some detailed information as to whether sentinel node mapping or systemic lymphadenectomy was used during the surgery and whether para-aortic LN sampling was performed had not been included in the SEER database, which might impact the assessment of NRLN. Since ratio-based LN schemes generally performed better than did number-based LN schemes, we believe that this would not significantly affect our results. Surely, more precise evaluations about LN schemes for node-positive EEC patients are welcome. Finally, the LNR ranged from 0 to 1, and the LODDS varied from negative infinity to positive infinity theoretically, but in the current study, we were confined to the setting of −4.07 < LODDS ≤4.32 and 0.01 ≤ LNR ≤1. As a result, it remained unclear whether the values exceeding these ranges had the same predictive performance.

## Conclusion

The AJCC N staging system (N1/N2) could not differentiate the prognosis for node-positive EEC patients in our study, which might suggest that a more aggressive adjuvant therapy was not necessary for N2 EEC patients than N1 patients. Moreover, we firstly established the prognostic role of the LODDS for node-positive EEC patients. LODDS had superior discrimination power than other LN schemes in continuous pattern, but LNR performed best in categorical pattern—both of which should be more significant in predicting the survival outcomes for node-positive EEC patients than AJCC N stage.

## Data Availability Statement

The datasets presented in this study can be found in online repositories. The names of the repository/repositories and accession number(s) can be found at: SEER.

## Author Contributions

X-LY: conception or design and manuscript writing. X-LY and M-MW: provision of study material or patients. X-LY and NH: collection and/or assembly of data. X-LY, NH, and M-MW: data analysis and interpretation. HL and D-JW: manuscript revision. All authors contributed to the article and approved the submitted version.

## Conflict of Interest

The authors declare that the research was conducted in the absence of any commercial or financial relationships that could be construed as a potential conflict of interest.
