# Supplementary material for: Comparison of Different Lymph Node Staging Schemes for Predicting Survival Outcomes in Node-Positive Endometrioid Endometrial Cancer Patients
Source: Front Med (Lausanne). 2021 Jul 9;8:688535. doi: 10.3389/fmed.2021.688535 (PMC8298894; doi:10.3389/fmed.2021.688535)
Supplement: Supplementary file 1 [file Data_Sheet_1.docx]

| **Table.S1** The selection process for eligible patients. | | | | |
| --- | --- | --- | --- | --- |
| Steps | Criteria | 1. of excluded | | N. of remaining patients |
| 1 | Patients diagnosed with EEC between 2010 and 2016 | - | 92868 | |
| 2 | Patients with insufficient informations | 1381 | 91487 | |
| 3 | Patients without LN metastasis (N0 stage) | 83171 | 8316 | |
| 4 | Patients without LN retrieving during the surgery | 1688 | 6628 | |
| 5 | Patients with T4 or M1 stage | 2570 | 4058 | |
| 6 | Patients without hysterectomy performing | 222 | 3836 | |
| 7 | Patients died from surgical complications (survival time less than 1 month) | 72 | 3764 | |
| 8 | Patients aged less than 18 years old | 3 | 3761 | |
| 8 | Patients with malignant tumor history | 225 | 3533 | |

EEC: Endometrioid endometrial cancer; LN: Lymph node.

| **Table.S2** Univariable Cox regression analysis of potential prognostic predictors for OS and DSS. | | | | | |
| --- | --- | --- | --- | --- | --- |
|  | OS | |  | DSS | |
| Variable | HR (95% CI) | *P*-value |  | HR (95% CI) | *P*-value |
| **Age** |  |  |  |  |  |
| <65 | 1 |  |  | 1 |  |
| ≥65 | 1.955 (1.722, 2.218) | *P*<0.001 |  | 1.951 (1.707, 2.230) | *P*<0.001 |
| **Race** |  |  |  |  |  |
| White | 1 |  |  | 1 |  |
| Black | 1.578 (1.330, 1.872) | *P*<0.001 |  | 1.612 (1.345, 1.931) | *P*<0.001 |
| Other | 0.838 (0.673, 1.044) | *P*=0.116 |  | 0.881 (0.702, 1.106) | *P*=0.275 |
| **Marital status** |  |  |  |  |  |
| Married | 1 |  |  | 1 |  |
| Unmarried | 1.348 (1.189, 1.528) | *P*<0.001 |  | 1.317 (1.153, 1.504) | *P*<0.001 |
| **Grade** |  |  |  |  |  |
| Ⅰ | 1 |  |  | 1 |  |
| Ⅱ | 1.225 (0.932, 1.611) | *P*=0.146 |  | 1.262 (0.938, 1.697) | *P*=0.125 |
| Ⅲ | 3.442 (2.691, 4.404) | *P*<0.001 |  | 3.705 (2.837, 4.837) | *P*<0.001 |
| Ⅳ | 3.947 (3.022, 5.155) | *P*<0.001 |  | 4.226 (3.170, 5.634) | *P*<0.001 |
| **T stage** |  |  |  |  |  |
| T1 | 1 |  |  | 1 |  |
| T2 | 1.481 (1.238, 1.773) | *P*<0.001 |  | 1.562 (1.293, 1.888) | *P*<0.001 |
| T3 | 2.605 (2.266, 2.994) | *P*<0.001 |  | 2.721 (2.346, 3.155) | *P*<0.001 |
| **Tumor size** |  |  |  |  |  |
| <4cm | 1 |  |  | 1 |  |
| ≥4cm | 1.411 (1.201, 1.657) | *P*<0.001 |  | 1.383 (1.169, 1.636) | *P*<0.001 |
| Unknown | 1.151 (0.935, 1.416) | *P*=0.185 |  | 1.094 (0.878, 1.364) | *P*=0.424 |
| **Surgery type** |  |  |  |  |  |
| Total | 1 |  |  | 1 |  |
| Radical | 1.326 (1.129, 1.558) | *P*<0.001 |  | 1.351 (1.140, 1.602) | *P*<0.001 |
| **Radiation** |  |  |  |  |  |
| Yes | 1 |  |  | 1 |  |
| No | 1.841 (1.624, 2.087) | *P*<0.001 |  | 1.799 (1.576, 2.054) | *P*<0.001 |
| **Chemo** |  |  |  |  |  |
| Yes | 1 |  |  | 1 |  |
| No/Unknown | 1.587 (1.384, 1.820) | *P*<0.001 |  | 1.524 (1.316, 1.765) | *P*<0.001 |
| **N stage** |  |  |  |  |  |
| N1 | 1 |  |  | 1 |  |
| N2 | 1.296 (1.143, 1.470) | *P*<0.001 |  | 1.362 (1.192, 1.555) | *P*<0.001 |
| **NRLN** |  |  |  |  |  |
| NRLN1 | 1 |  |  | 1 |  |
| NRLN2 | 0.588 (0.488, 0.709) | *P*<0.001 |  | 0.585 (0.480, 0.713) | *P*<0.001 |
| NRLN3 | 0.485 (0.408, 0.577) | *P*<0.001 |  | 0.492 (0.410, 0.591) | *P*<0.001 |
| **NPLN** |  |  |  |  |  |
| NPLN1 | 1 |  |  | 1 |  |
| NPLN2 | 1.291 (1.120, 1.488) | *P*<0.001 |  | 1.325 (1.140, 1.540) | *P*<0.001 |
| NPLN3 | 2.394 (1.997, 2.869) | *P*<0.001 |  | 2.453 (2.024, 2.972) | *P*<0.001 |
| **NNLN** |  |  |  |  |  |
| NNLN1 | 1 |  |  | 1 |  |
| NNLN2 | 0.576 (0.491, 0.675) | *P*<0.001 |  | 0.575 (0.486, 0.681) | *P*<0.001 |
| NNLN3 | 0.395 (0.338, 0.461) | *P*<0.001 |  | 0.395 (0.335, 0.466) | *P*<0.001 |
| **LNR** |  |  |  |  |  |
| LNR1 | 1 |  |  | 1 |  |
| LNR2 | 1.761 (1.534, 2.023) | *P*<0.001 |  | 1.778 (1.535, 2.059) | *P*<0.001 |
| LNR3 | 3.828 (3.205, 4.572) | *P*<0.001 |  | 3.978 (3.300, 4.795) | *P*<0.001 |
| **LODDS** |  |  |  |  |  |
| LODDS1 | 1 |  |  | 1 |  |
| LODDS2 | 1.778 (1.548, 2.042) | *P*<0.001 |  | 1.781 (1.538, 2.063) | *P*<0.001 |
| LODDS3 | 3.750 (3.149, 4.464) | *P*<0.001 |  | 3.889 (3.238, 4.671) | *P*<0.001 |

OS: Overall survival; DSS: Disease-specific survival; NRLN: Number of retrieved lymph node; NPLN: Number of positive lymph node; NNLN: Number of

negative lymph node; LNR: Lymph node ratio; LODDS: Log odds of positive lymph node; CI: Confidence interval.

| **Table.S3** Multivariable Cox regression analysis (Model 1–6) of prognostic predictors for DSS. | | | | | | | | | | | | | | | | | |
| --- | --- | --- | --- | --- | --- | --- | --- | --- | --- | --- | --- | --- | --- | --- | --- | --- | --- |
|  | Model1 (N) | | | Model2 (NRLN) | | | Model3 (NPLN) | | | Model4 (NNLN) | | | Model5 (LNR) | | | Model6 (LODDS) | |
| Variable | HR (95% CI) | *P*-value |  | HR (95% CI) | *P*-value |  | HR (95% CI) | *P*-value |  | HR (95% CI) | *P*-value |  | HR (95% CI) | *P*-value |  | HR (95% CI) | *P*-value |
| **Age** |  |  |  |  |  |  |  |  |  |  |  |  |  |  |  |  |  |
| <65 | 1 |  |  | 1 |  |  | 1 |  |  | 1 |  |  | 1 |  |  | 1 |  |
| ≥65 | 1.664 (1.451, 1.909) | *P*<0.001 |  | 1.688 (1.472, 1.936) | *P*<0.001 |  | 1.662 (1.449, 1.906) | *P*<0.001 |  | 1.688 (1.471, 1.936) | *P*<0.001 |  | 1.699 (1.481, 1.949) | *P*<0.001 |  | 1.696 (1.479, 1.945) | *P*<0.001 |
| **Race** |  |  |  |  |  |  |  |  |  |  |  |  |  |  |  |  |  |
| White | 1 |  |  | 1 |  |  | 1 |  |  | 1 |  |  | 1 |  |  | 1 |  |
| Black | 1.223 (1.016, 1.472) | *P=*0.033 |  | 1.213 (1.006, 1.461) | *P=*0.043 |  | 1.195 (0.992, 1.439) | *P=*0.060 |  | 1.157 (0.960, 1.395) | *P=*0.126 |  | 1.123 (0.931, 1.353) | *P=*0.227 |  | 1.125 (0.933, 1.357) | *P=*0.217 |
| Other | 0.895 (0.711, 1.126) | *P=*0.343 |  | 0.888 (0.706, 1.118) | *P=*0.313 |  | 0.914 (0.726, 1.151) | *P=*0.444 |  | 0.875 (0.695, 1.102) | *P=*0.258 |  | 0.876 (0.700, 1.103) | *P=*0.260 |  | 0.877 (0.696, 1.104) | *P=*0.264 |
| **Marital status** |  |  |  |  |  |  |  |  |  |  |  |  |  |  |  |  |  |
| Married | 1 |  |  | 1 |  |  | 1 |  |  | 1 |  |  | 1 |  |  | 1 |  |
| Unmarried | 1.094 (0.955, 1.254) | *P=*0.195 |  | 1.079 (0.941, 1.237) | *P=*0.274 |  | 1.097 (0.957, 1.258) | *P=*0.185 |  | 1.075 (0.937, 1.232) | *P=*0.303 |  | 1.092 (0.952, 1.252) | *P=*0.209 |  | 1.084 (0.945, 1.242) | *P=*0.251 |
| **Grade** |  |  |  |  |  |  |  |  |  |  |  |  |  |  |  |  |  |
| Ⅰ | 1 |  |  | 1 |  |  | 1 |  |  | 1 |  |  | 1 |  |  | 1 |  |
| Ⅱ | 1.266 (0.940, 1.704) | *P=*0.121 |  | 1.284 (0.953, 1.729) | *P=*0.100 |  | 1.242 (0.923, 1.673) | *P=*0.153 |  | 1.284 (0.953, 1.730) | *P=*0.100 |  | 1.268 (0.942, 1.707) | *P=*0.118 |  | 1.247 (0.926, 1.679) | *P=*0.146 |
| Ⅲ | 2.916 (2.221, 3.829) | *P*<0.001 |  | 2.919 (2.223, 3.833) | *P*<0.001 |  | 2.826 (2.151, 3.713) | *P*<0.001 |  | 2.874 (2.189, 3.773) | *P*<0.001 |  | 2.778 (2.115, 3.650) | *P*<0.001 |  | 2.750 (2.093, 3.613) | *P*<0.001 |
| Ⅳ | 3.073 (2.290, 4.124) | *P*<0.001 |  | 3.161 (2.354, 4.244) | *P*<0.001 |  | 2.976 (2.217, 3.997) | *P*<0.001 |  | 3.129 (2.331, 4.200) | *P*<0.001 |  | 3.053 (2.275, 4.098) | *P*<0.001 |  | 3.017 (2.248, 4.050) | *P*<0.001 |
| **T stage** |  |  |  |  |  |  |  |  |  |  |  |  |  |  |  |  |  |
| T1 | 1 |  |  | 1 |  |  | 1 |  |  | 1 |  |  | 1 |  |  | 1 |  |
| T2 | 1.316 (1.087, 1.594) | *P=*0.005 |  | 1.315 (1.086, 1.592) | *P=*0.005 |  | 1.299 (1.072, 1.574) | *P=*0.008 |  | 1.288 (1.063, 1.559) | *P=*0.010 |  | 1.276 (1.054, 1.546) | *P=*0.013 |  | 1.281 (1.058, 1.552) | *P=*0.011 |
| T3 | 2.190 (1.873, 2.561) | *P*<0.001 |  | 2.136 (1.828, 2.496) | *P*<0.001 |  | 2.074 (1.771, 2.430) | *P*<0.001 |  | 2.025 (1.730, 2.370) | *P*<0.001 |  | 1.844 (1.570, 2.166) | *P*<0.001 |  | 1.843 (1.569, 2.164) | *P*<0.001 |
| **Tumor size** |  |  |  |  |  |  |  |  |  |  |  |  |  |  |  |  |  |
| <4cm | 1 |  |  | 1 |  |  | 1 |  |  | 1 |  |  | 1 |  |  | 1 |  |
| ≥4cm | 1.217 (1.026, 1.443) | *P=*0.024 |  | 1.204 (1.015, 1.428) | *P=*0.033 |  | 1.196 (1.008, 1.419) | *P=*0.040 |  | 1.184 (0.998, 1.404) | *P=*0.053 |  | 1.191 (1.004, 1.413) | *P=*0.045 |  | 1.188 (1.001, 1.409) | *P=*0.049 |
| Unknown | 1.010 (0.809, 1.261) | *P=*0.931 |  | 1.027 (0.822, 1.283) | *P=*0.815 |  | 0.988 (0.791, 1.235) | *P=*0.918 |  | 1.015 (0.813, 1.268) | *P=*0.893 |  | 0.969 (0.776, 1.211) | *P=*0.783 |  | 0.975 (0.780, 1.218) | *P=*0.821 |
| **Surgery type** |  |  |  |  |  |  |  |  |  |  |  |  |  |  |  |  |  |
| Total | 1 |  |  | 1 |  |  | 1 |  |  | 1 |  |  | 1 |  |  | 1 |  |
| Radical | 1.209 (1.018, 1.436) | *P=*0.030 |  | 1.231 (1.036, 1.463) | *P=*0.018 |  | 1.202 (1.012, 1.429) | *P=*0.036 |  | 1.246 (1.048, 1.481) | *P=*0.013 |  | 1.223 (1.029, 1.453) | *P=*0.022 |  | 1.227 (1.033, 1.458) | *P=*0.020 |
| **Radiation** |  |  |  |  |  |  |  |  |  |  |  |  |  |  |  |  |  |
| Yes | 1 |  |  | 1 |  |  | 1 |  |  | 1 |  |  | 1 |  |  | 1 |  |
| No | 1.540 (1.340, 1.770) | *P*<0.001 |  | 1.524 (1.327, 1.751) | *P*<0.001 |  | 1.519 (1.322, 1.747) | *P*<0.001 |  | 1.527 (1.329, 1.754) | *P*<0.001 |  | 1.476 (1.284, 1.697) | *P*<0.001 |  | 1.482 (1.289, 1.704) | *P*<0.001 |
| **Chemo** |  |  |  |  |  |  |  |  |  |  |  |  |  |  |  |  |  |
| Yes | 1 |  |  | 1 |  |  | 1 |  |  | 1 |  |  | 1 |  |  | 1 |  |
| No/Unknown | 1.461 (1.249,1.708) | *P*<0.001 |  | 1.451 (1.242, 1.697) | *P*<0.001 |  | 1.471 (1.258, 1.720) | *P*<0.001 |  | 1.437 (1.229, 1.680) | *P*<0.001 |  | 1.480 (1.265, 1.730) | *P*<0.001 |  | 1.475 (1.261, 1.725) | *P*<0.001 |
| **N stage** |  |  |  |  |  |  |  |  |  |  |  |  |  |  |  |  |  |
| N1 | 1 |  |  |  |  |  |  |  |  |  |  |  |  |  |  |  |  |
| N2 | 1.106 (0.966, 1.267) | *P=*0.145 |  |  |  |  |  |  |  |  |  |  |  |  |  |  |  |
| **NRLN** |  |  |  |  |  |  |  |  |  |  |  |  |  |  |  |  |  |
| NRLN1 |  |  |  | 1 |  |  |  |  |  |  |  |  |  |  |  |  |  |
| NRLN2 |  |  |  | 0.665 (0.544, 0.813) | *P*<0.001 |  |  |  |  |  |  |  |  |  |  |  |  |
| NRLN3 |  |  |  | 0.600 (0.498, 0.722) | *P*<0.001 |  |  |  |  |  |  |  |  |  |  |  |  |
| **NPLN** |  |  |  |  |  |  |  |  |  |  |  |  |  |  |  |  |  |
| NPLN1 |  |  |  |  |  |  | 1 |  |  |  |  |  |  |  |  |  |  |
| NPLN2 |  |  |  |  |  |  | 1.157 (0.993, 1.348) | *P=*0.061 |  |  |  |  |  |  |  |  |  |
| NPLN3 |  |  |  |  |  |  | 1.598 (1.308, 1.952) | *P*<0.001 |  |  |  |  |  |  |  |  |  |
| **NNLN** |  |  |  |  |  |  |  |  |  |  |  |  |  |  |  |  |  |
| NNLN1 |  |  |  |  |  |  |  |  |  | 1 |  |  |  |  |  |  |  |
| NNLN2 |  |  |  |  |  |  |  |  |  | 0.651 (0.549, 0.773) | *P*<0.001 |  |  |  |  |  |  |
| NNLN3 |  |  |  |  |  |  |  |  |  | 0.514 (0.434, 0.610) | *P*<0.001 |  |  |  |  |  |  |
| **LNR** |  |  |  |  |  |  |  |  |  |  |  |  |  |  |  |  |  |
| LNR1 |  |  |  |  |  |  |  |  |  |  |  |  | 1 |  |  |  |  |
| LNR2 |  |  |  |  |  |  |  |  |  |  |  |  | 1.503 (1.293, 1.746） | *P*<0.001 |  |  |  |
| LNR3 |  |  |  |  |  |  |  |  |  |  |  |  | 2.656 (2.176, 3.241) | *P*<0.001 |  |  |  |
| **LODDS** |  |  |  |  |  |  |  |  |  |  |  |  |  |  |  |  |  |
| LODDS1 |  |  |  |  |  |  |  |  |  |  |  |  |  |  |  | 1 |  |
| LODDS2 |  |  |  |  |  |  |  |  |  |  |  |  |  |  |  | 1.495 (1.287, 1.737) | *P*<0.001 |
| LODDS3 |  |  |  |  |  |  |  |  |  |  |  |  |  |  |  | 2.594 (2.133, 3.155) | *P*<0.001 |

DSS: Disease-specific survival; NRLN: Number of retrieved lymph node; NPLN: Number of positive lymph node; NNLN: Number of negative lymph node; LNR: Lymph node ratio; LODDS: Log odds of positive lymph node; CI: Confidence interval.


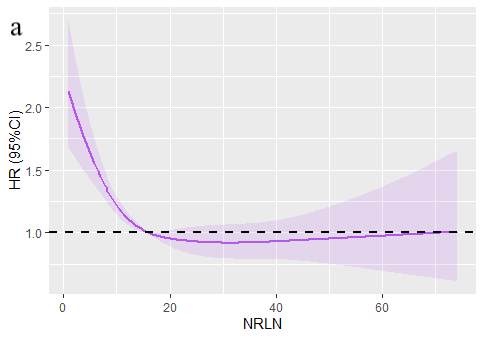

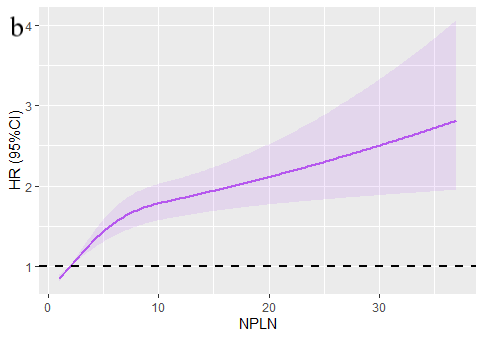


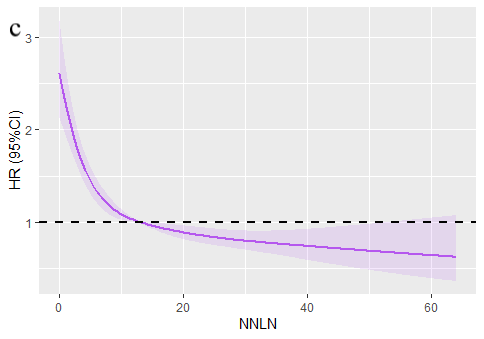

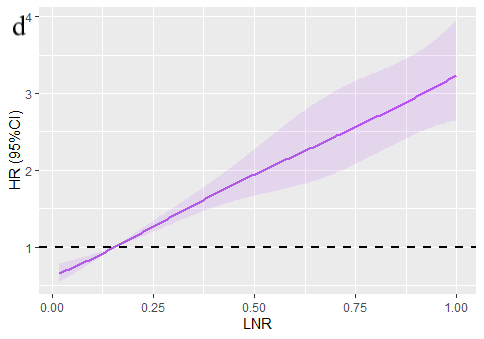

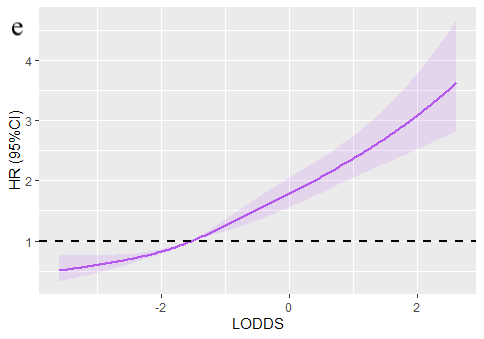


**Fig.S1** HR as a function of NRLN (a), NPLN (b), NNLN (c), LNR (d) and LODDS (e) for the whole study cohort. 95% CI (Confidence interval) was indicated.HR: Hzard ratio; NRLN: Number of retrieved lymph node; NPLN: Number of positive

lymph node; NNLN: Number of negative lymph node; LNR: Lymph node ratio; LODDS: Log odds of positive lymph node.


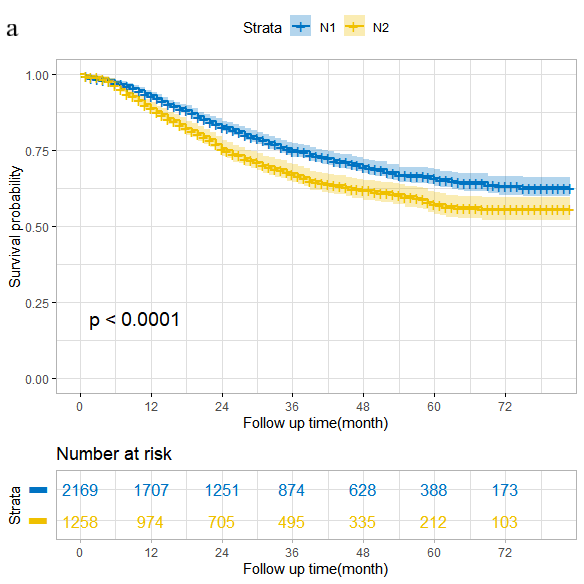

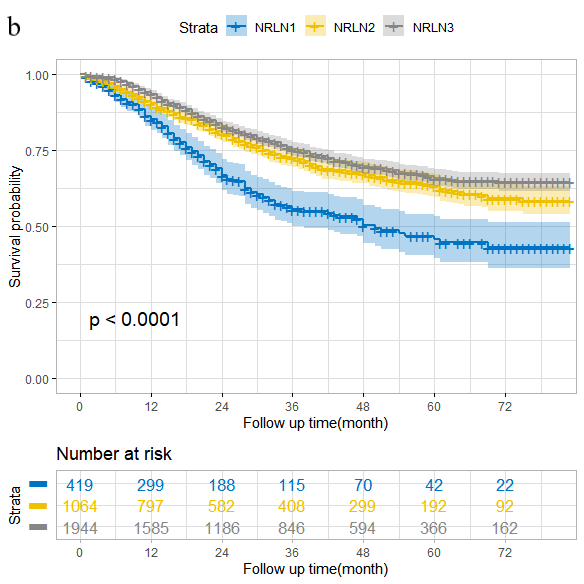

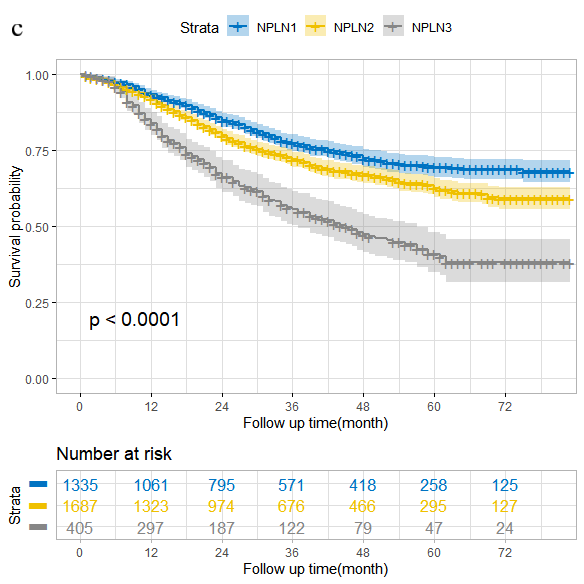


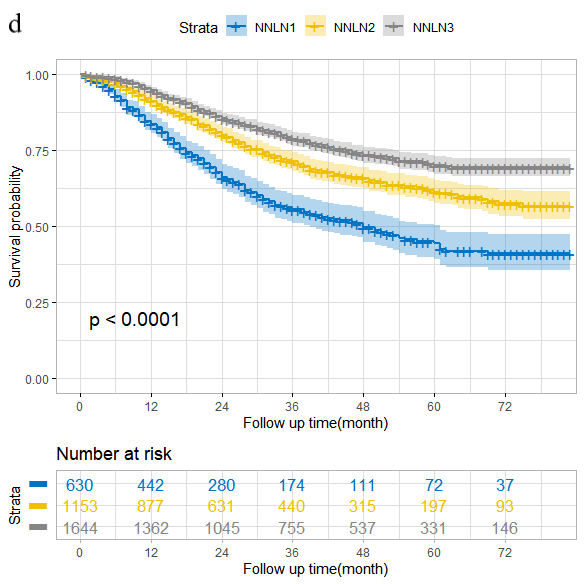

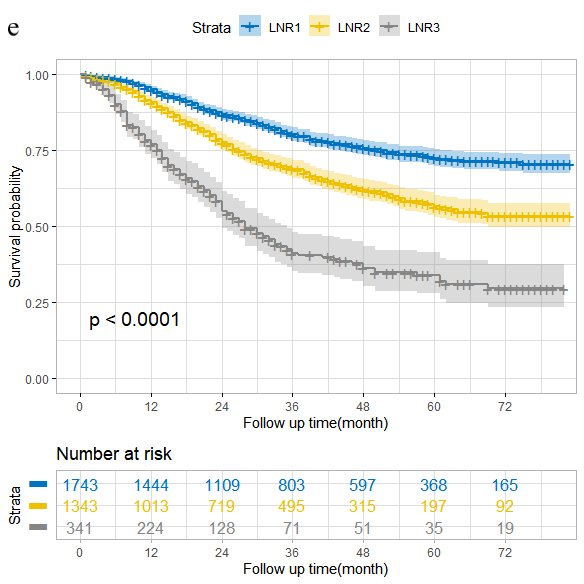

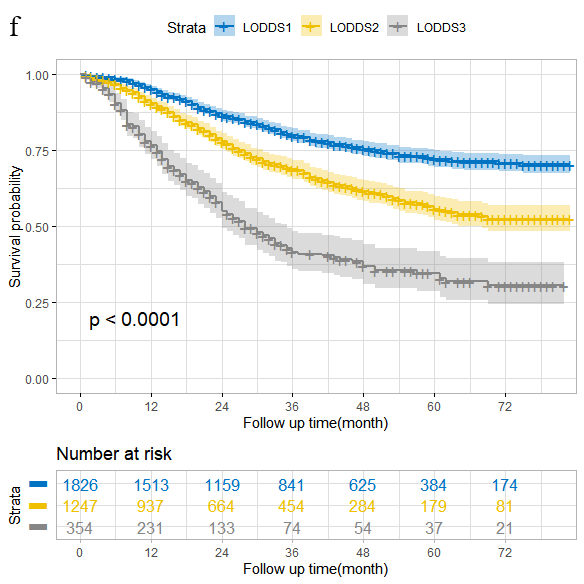


**Fig.S2** Kaplan-Meier estimates of disease-specific survival according to (a) N, (b) NRLN, (c) NPLN, (d) NNLN, (e) LNR and (f) LODDS staging systems. NRLN: Number of retrieved lymph node;

NPLN: Number of positive lymph node; NNLN: Number of negative lymph node; LNR: Lymph node ratio; LODDS: Log odds of positive lymph node.
